# Association of emotional and behavioral problems with the development of the substantia nigra, subthalamic nucleus, and red nucleus volumes and asymmetries from childhood to adolescence: A longitudinal cohort study

**DOI:** 10.1038/s41398-024-02803-4

**Published:** 2024-02-26

**Authors:** Yanpei Wang, Leilei Ma, Jiali Wang, Ningyu Liu, Weiwei Men, Shuping Tan, Jia-Hong Gao, Shaozheng Qin, Yong He, Qi Dong, Sha Tao

**Affiliations:** 1 State Key Laboratory of Cognitive Neuroscience and Learning, Beijing Normal University, Beijing, 100875 China; 2 IDG/McGovern Institute for Brain Research, Beijing Normal University, Beijing, 100875 China; 3 Center for MRI Research, Academy for Advanced Interdisciplinary Studies, Peking University, Beijing, 100871 China; 4 Psychiatry Research Center, Beijing HuiLongGuan Hospital, Peking University, Beijing, 100096 China

**Keywords:** Predictive markers, Human behaviour

## Abstract

The substantia nigra (SN), subthalamic nucleus (STN), and red nucleus (RN) have been widely studied as important biomarkers of degenerative diseases. However, how they develop in childhood and adolescence and are affected by emotional behavior has not been studied thus far. This population-based longitudinal cohort study used data from a representative sample followed two to five times. Emotional and behavioral problems were assessed with the Strengths and Difficulties Questionnaire (SDQ). Linear mixed models were used to map developmental trajectories and behavioral regulation. Using an innovative automated image segmentation technique, we quantified the volumes and asymmetries of the SN, STN and RN with 1226 MRI scans of a large longitudinal sample of 667 subjects aged 6–15 years and mapped their developmental trajectories. The results showed that the absolute and relative volumes of the bilateral SN and right STN showed linear increases, while the absolute volume of the right RN and relative volume of the bilateral RN decreased linearly, these effects were not affected by gender. Hyperactivity/inattention weakened the increase in SN volume and reduced the absolute volume of the STN, conduct problems impeded the RN volume from decreasing, and emotional symptoms changed the direction of SN lateralization. This longitudinal cohort study mapped the developmental trajectories of SN, STN, and RN volumes and asymmetries from childhood to adolescence, and found the association of emotional symptoms, conduct problems, and hyperactivity/inattention with these trajectories, providing guidance for preventing and intervening in cognitive and emotional behavioral problems.

## Introduction

The substantia nigra (SN), subthalamic nucleus (STN), and red nucleus (RN) are small and important subcortical nuclei in the basal ganglia, located in the midbrain that are involved in fundamental processes such as motor control, emotion and cognition [1]. Because they are directly next to each other, small and variable in their anatomical location [1], they are often studied together. These three structures are considered to be closely related to the dopamine system, SN is a midbrain dopaminergic nucleus [2], STN regulates the nigral dopamine neuron activity [3], and RN is thought to provide a compensatory mechanism for the damage involved in SN [4]. Most studies of these three structures have focused on neurodegenerative diseases such as Parkinson’s and Alzheimer’s disease [5–7]. In fact, the dopamine system also plays an important role in childhood and adolescence development, especially emotional behavior problems, such as ADHD [8] and depression [9, 10]. However, our knowledge regarding the maturation in the human brain from childhood into adolescence and how they are affected by emotional and behavioral problems is still very limited.

The SN is the most studied of the three structures. It is a midbrain dopaminergic nucleus that can modulate motor movement and reward functions and is classically considered to be the gate of the basal ganglia circuitry [2]. Approximately 68% of all dopaminergic neurons identified in the mesencephalon are found in the SN [11], which plays a major role in disease due to the degeneration of dopaminergic cells [12]. Whereas aging is associated with a significant decline in SN markers in the human brain, most studies have focused on adult subjects [5], and few studies have assessed the effect of age on the transition from childhood to adolescence. To our knowledge, a total of two studies have analyzed the developmental trajectory of SN echogenicity. One study reported a gradual decline in SN echogenicity after birth in 109 children aged 0–192 months [13], while the other study found no age effect on 44 samples aged 7–16 years [14]. However, since these two studies were based on cross-sectional samples and the sample sizes were small, it is difficult to draw accurate conclusions based on them with inconsistent results.

In addition to aging, the SN may be a stable biomarker for evaluating neuropsychiatric disorders, especially attention-deficit/hyperactivity disorder (ADHD) and emotional problems. Early studies on SN and ADHD adopted echogenicity, and found that ADHD individuals had larger echogenic SN areas [14], and echogenic size was positively correlated with ADHD symptoms [15], which was reaffirmed by a recent study using transcranial sonography [16]. Studies based on brain imaging have found that ADHD had higher functional connections between the SN and the amygdala and thalamus [8], as well as the striatum [17]. Second, an animal model showed that the SN could moderate the degeneration and apoptosis of dopaminergic neurons, which play a key role in preventing depressive symptom [9]. Based on a study of patients with traumatic brain injury, it was found that the connection between the SN and the left angular gyrus was positively correlated with post-traumatic anxiety symptoms and negatively correlated with depressive symptoms [10]. However, how emotional and behavioral problems affect SN structure development from childhood into adolescence remains unclear.

The STN is a small, glutamatergic nucleus situated in the diencephalon [18], which is located next to the SN, highly connected to the SN and part of the basal ganglia circuit that controls motor actions [12]. Due to the partial volume effect, the functional signal from the STN is very likely to be mixed with those from the SN [19]. Previous studies using fMRI found that the STN and SN are involved in a range of tasks such as response inhibition [20], conflict processing [21], working memory [22, 23], and task switching [24]. The function of the STN has been studied in more detail than that of the SN based on animal and clinical patient studies through other techniques, such as single neuron recording, deep brain stimulation (DBS) and event-related potentials (ERPs). By recording the single neuron activity of the STN in monkeys, researchers found that STN neurons were associated with reward and delayed reward during task performance [25]. A review of studies based on rats suggested that the STN plays an important role in neural circuits of behavioral inhibition, which is usually omitted from conventional behavioral-inhibition networks [26]. The DBS based on ERPs showed that auditory event attention involved the STN, which was significantly prior to the P3 cortical response [27]. A lesion study found that STN lesions could increase impulsive action (produced behavioral disinhibition) and decrease impulsive choice (impulsive decision making), indicating that the STN may serve as a novel target for the treatment of psychological diseases characterized by deficits in behavioral control, especially ADHD [28]. In general, the function of the STN involves mainly reward and inhibitory control, and the STN may become an important therapeutic target for ADHD; however, this has not been confirmed in humans.

The RN is also located close to the SN and is involved in motor control through its connections with the cerebellum and the cortical sensorimotor cortex [4]. The RN, along with the cerebellum and thalamus, forms the thalamo-rubro cerebellar pathway, which plays an important role in the neural compensatory mechanism of neurodegenerative diseases [4]. For example, one study found that by the time patients were diagnosed, the loss of dopamine cells in the SN had reached 60 percent or more, yet the patient showed no motor symptoms before that [29]. Philippens et al. demonstrated in marmosets that this is a compensatory mechanism, they found that when SN dopamine-producing cells are loss, RN activity increases and compensates for the dysfunction of the striato-thalamo-cortical circuit through the thalamo-rubro-cerebellar pathway [4]. The SN exports excitatory dopamine to the basal ganglia, which in turn acts on the striato-thalamo-cortical pathway. Therefore, the postmortem brains of Parkinson patients showed a 32% increase in the size of RN compared with healthy controls [30]. However, the compensation of RN for SN structure loss mainly occurred in Parkinson’s disease patients, and whether such compensation also exists during the development of children and adolescents remains unclear.

In addition, SN asymmetry was found to be an important pathological indicator of Parkinson’s disease, with differences in all diffusion parameters located on the left side [31]. One study based on echogenicity of the SN in childhood also found that the rate of decline was greater on the right side than the left [13]. However, no studies have been conducted to further investigate the developmental characteristics of SN asymmetry and its association with emotional and behavioral problems during development. So far, there is a paucity of research on the developmental trajectories of the RN and STN; however, given their close association with the SN, further investigation into their lateralization development is warranted.

Thus, our first aim was to delineate the developmental trajectories of the SN, STN, and RN from childhood to adolescence. Second, we sought to explore how emotional and behavioral problems affect the developmental trajectories of the SN, STN, and RN and ultimately lead to neurodevelopmental disorders associated with these subcortical structures, providing evidence to support the understanding of the biological mechanisms underlying behavior. We used a novel automated segmentation pipeline applied to 1226 MRI scans from 667 children and adolescents aged 6–15 years; drew the development trajectories of the absolute volume, relative volume and asymmetry of the SN, STN, and RN; and investigated the association of emotional and behavioral problems with their developmental trajectories from five aspects: emotional symptoms, conduct problems, hyperactivity/inattention, peer problems, and prosocial behaviors.

## Materials and methods

### Participants

We used a longitudinal structural MRI dataset comprising the data of 766 normally developing children aged 6–15 years (F/M = 334/432) from the Children School Functions and Brain Development project (CBD, Beijing Cohort). Participants underwent 2–4 repeated MRI scans at approximately one-year intervals, resulting in 1603 structural MRI scans in total. After quality control, a total of 667 children with 1226 MRI scans were used in this study. Sample selection and age distribution are included in the Supplementary Methods. All children’s parents/guardians signed an informed consent form approved by the Ethics Committee of Beijing Normal University.

### Assessments of emotional and behavioral problems

The parent-reported version of the Strengths and Difficulties Questionnaire (SDQ) was applied to assess emotional and behavioral problems in the children and adolescents, and was collected at every visit. The Chinese version was retrieved from the SDQ website (https://www.sdqinfo.org/py/sdqinfo/b0.py), which includes multiple language versions. The SDQ included five subscales (emotional symptoms, conduct problems, hyperactivity or peer problems, and a prosocial behavior scale) comprising 25 items [32].

### Image acquisition

All structural T2 images were obtained at Peking University, Huilongguan Hospital and Beijing Normal University, Beijing. Three identical Siemens Prisma 3T MRI scanners with 64-channel head coils and the same parameters were used at the three sites. T2-weighted anatomical scans with the following parameters were obtained at each time point: 3D T2-SPACE sequence, TR = 3200 ms, TE = 564 ms, acquisition matrix = 320 × 320, FOV = 224 × 224 mm^2^, slices = 256, in-plane resolution = 0.7 × 0.7 mm^2^, slice thickness = 0.7 mm, bandwidth = 744 Hz/Px, and scan time = 8 min and 24 s. The MRI quality control process is presented in the Supplementary Methods.

### Data preprocessing

**Fig. 1:**
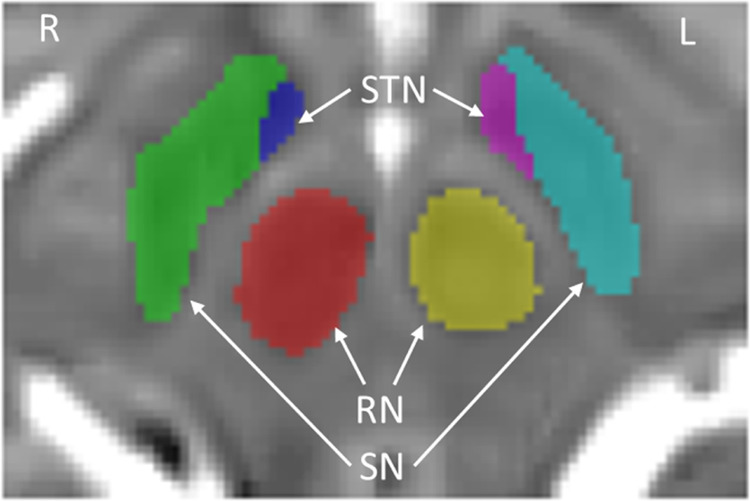
The segmentation of the SN, STN, and RN. SN substantia nigra, STN subthalamic nucleus, RN red nucleus, L left, and R right.

We computed the volumes of the SN, STN and RN using the automated software pipeline pBrain (https://www.volbrain.upv.es) [12]. Briefly, the pBrain segmentation pipeline of each T2w image was as follows: First, the high-resolution image was registered to MNI152 space, with inhomogeneity corrected and intensity normalized. Later, the image was cropped and denoised and the case-specific library was constructed. Finally, the OPAL (Optimized PAtchMatch Label fusion) method was used to produce an initial segmentation which was later refined using the patch-based ensemble corrector method [33]. After preprocessing, pBrain produced the volumes of the SN, STN and RN as outputs, as shown in Fig. 1. This study will use absolute and relative volume, absolute volume refers to the volume directly measured by pBrain, while relative volume refers to the relative proportion of absolute volume to intracranial volume. The asymmetry index is calculated as the difference between the right and left volumes divided by their average, with larger values indicating stronger right lateralization [34].

### Statistical analysis

We performed statistical analyses using R 4.1.1 (https://www.r-project.org/). To investigate the developmental trajectories of the volumes of the SN, STN and RN and the effects of sex, we used mixed models with the nlme package [35].

Mixed modeling approaches are very suitable for accelerated longitudinal designs and can deal with missing data; hence, they are widely used [36]. Each dependent measure of the i^th^ family, *j*^th^ individual and k^th^ time-point was modeled, and detailed information was described by Raznahan et al. [37]. We fitted several models, including linear age terms, quadratic age terms, cubic age terms and gender terms. The following equation shows the full model:

$$\begin{array}{c}{{\rm{Measurement}}}_{{\rm{ijk}}}={\rm{Intercept}}+{{\rm{d}}}_{{\rm{ij}}}+{\beta }_{0}({\rm{site}})+{\beta }_{1}({\rm{gender}})+{\beta }_{2}({\rm{age}})+{\beta }_{3}({{\rm{age}}}^{2})\\ \,+\,{\beta }_{4}({{\rm{age}}}^{3})+{\beta }_{3}({\rm{gender}}\times {\rm{age}})+{\beta }_{6}({\rm{gender}}\times {{\rm{age}}}^{2})\\ \,+\,{\beta }_{7}({\rm{gender}}\times {{\rm{age}}}^{3})+{{\rm{e}}}_{{\rm{ijk}}}\end{array}$$

The e_ijk_ term represents the residual error of the normal distribution. Each β represents a parameter estimate; for example, the quadratic age effect parameter is represented by β_3_. Furthermore, the interaction effects of gender and age were modeled. The full model was compared with models including only linear or quadratic cubic age terms. Intercept, gender and age were fixed effects, while within-person dependence nested within subject (d_ij_) was modeled as a random effect.

The formal model-fitting procedure was applied to all mixed models. Preferred models were identified based on their lower Bayesian Information Criterion (BIC) values, indicating a superior fit to the data compared to alternative models. The comparison of BIC values follows a rule of thumb. Generally, Model X is considered more suitable for data than Model Y, where the BIC of Model X decreases by more than 1+k, where k represents the number of additional parameters [38]. First, an unconditional means model was employed, incorporating both fixed and random intercepts account for individual differences. Second, these models were compared with three commonly used growth models that examined the overall trajectory of age using a polynomial function [39]. Third, a random slope was added to the best-fitting age model to assess whether this improved model fit. Fourth, to investigate sex differences change over time, we added sex as a main effect and an interaction effect respectively to the best-fitting model and tested whether either of these improved model fit.

Then, we investigated how children’s emotional and behavioral problems affect the development of the volumes of the SN, STN and RN. Then, we added this continuous emotional and behavioral problems score (centered) to the best-fitting mixed model and inspected the significance of its main and age interaction terms. To visualize and better show the role of emotional and behavioral problems, the sample was split into two subgroups, as detailed in Supplemental Methods.

Finally, to optimally balance between Type-I and Type-II errors, we took the correlation between the dependent variables into account by using an adjusted Bonferroni procedure [40, 41], leading to an equivalent corrected alpha of 0.0127 for absolute volumes, 0.0119 for relative volumes, and 0.0214 for asymmetries, as detailed in the Supplemental Methods.

## Results

### Developmental trajectories

**Fig. 2:**
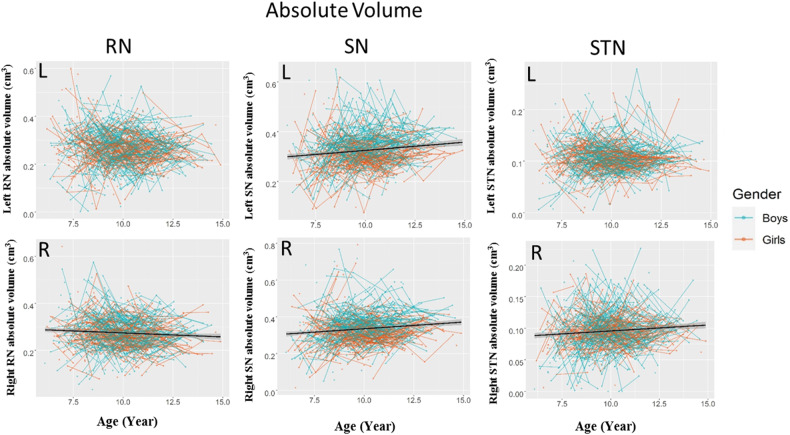
Development of RN, SN, and STN absolute volumes. The shaded areas represent the 95%confidence intervals. Individual boys (blue) and girls (orange) are represented by individual lines, and participants measured once are represented by dots. RN red nucleus, SN substantia nigra, STN subthalamic nucleus.

**Fig. 3:**
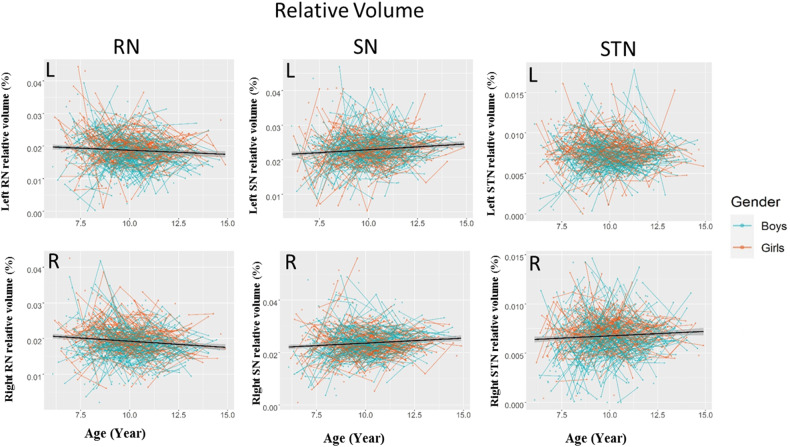
Development of RN, SN, and STN relative volumes. The shaded areas represent the 95%confidence intervals. Individual boys (blue) and girls (orange) are represented by individual lines, and participants measured once are represented by dots. RN red nucleus, SN substantia nigra, STN subthalamic nucleus.

**Fig. 4:**
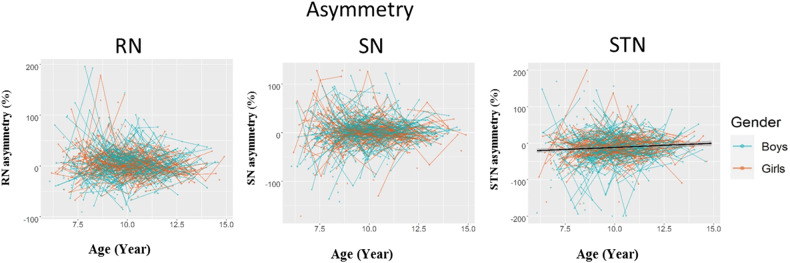
Development of RN, SN, and STN asymmetries. The shaded areas represent the 95%confidence intervals. Individual boys (blue) and girls (orange) are represented by individual lines, and participants measured once are represented by dots. RN red nucleus, SNsubstantia nigra, STN subthalamic nucleus.

BIC values for the different unconditional means models and age models for the absolute and relative volumes of the SN, STN and RN are reported in Table S1.The results of the regression models for the absolute and relative volumes of the bilateral SN, STN and RN are shown in Figs. 2–4 and Table 1. The absolute and relative volumes of the bilateral SN (absolute: left: *standardized β* = 0.130, *p* = 9.10 × 10^−6^, right: *standardized β* = 0.124, *p* = 2.19 × 10^−5^; relative: left: *standardized β* = 0.099, *p* = 9.30 × 10^−4^, right: *standardized β* = 0.096, *p* = 1.34 × 10^−3^) and right STN (absolute: *standardized β* = 0.107, *p* = 3.53 × 10^−4^; relative: *standardized β* = 0.078, *p* = 8.34 × 10^−3^) and the asymmetry of the STN (*standardized β* = 0.099, *p* = 9.04 × 10^−4^) showed linear growth, while the absolute volume of the right RN (*standardized β* = −0.0838, *p* = 4.92 × 10^−3^) and relative volume of the bilateral RN (left: *standardized β* = −0.077, *p* = 9.89 × 10^−3^, right: *standardized β* = −0.122, *p* = 4.02 × 10^−5^) decreased linearly. Since no significant interaction between sex and age was found, indicating that sex did not affect the developmental trajectories of the SN, RN and STN, gender was not considered in the subsequent analyses.

**Table 1:** Best fit regression model for absolute, relative volumes, asymmetry of the RN, SN, STN and parameters for developmental trajectories.

Volumes			Best fitting model	Intercept (s.e.)	Site coefficient *β*_1_ (s.e.)	Gender coefficient *β*_2_ (s.e.)	Age coefficient *β*_3_ (s.e.)	Age×gender coeffiicent *β*_4_ (s.e.)
Absolute volumes	RN	L	None	–	–	–	–	–
		R	Linear	3.12 (0.14) × 10^−1^	n.s.	–	−3.67 (1.30) × 10^−3^	–
	SN	L	Linear	2.56 (0.15) × 10^−1^	n.s.	2.33 (0.49) × 10^−2^	6.17 (1.38) × 10^−3^	–
		R	Linear	2.65 (0.18) × 10^−1^	n.s.	2.85 (0.61) × 10^−2^	6.97 (1.64) × 10^−3^	–
	STN	L	None	–	-	–	–	–
		R	Linear	7.92 (0.62) × 10^−2^	n.s.	–	2.01 (0.56) × 10^−3^	–
Relative volumes	RN	L	Linear	2.21 (0.11) × 10^−2^	n.s.	−1.39 (0.34) × 10^−3^	−2.54 (0.98) × 10^−4^	–
		R	Linear	2.37 (0.10) × 10^−2^	n.s.	−1.36 (0.33) × 10^−3^	−3.66 (0.89) × 10^−4^	–
	SN	L	Linear	2.00 (0.10) × 10^−2^	n.s.	–	3.10 (0.92) × 10^−4^	–
		R	Linear	2.09 (0.12) × 10^−2^	n.s.	–	3.61 (1.12) × 10^−4^	–
	STN	L	None	–	–	–	–	–
		R	Linear	6.22 (0.43) × 10^−3^	n.s.	−4.89 (1.33) × 10^−4^	1.01 (0.38) × 10^−4^	–
Asymmetries	RN		None	–	–	–	–	–
	SN		None	–	–	–	–	–
	STN		Linear	−3.63 (0.84) × 10	n.s.	–	2.53 (0.76)	

The level of significance is 0.0127 for absolute volumes, 0.0119 for relative volumes and 0.0214 for asymmetry after multiple comparisons correction, *RN* red nucleus, *SN* substantia nigra, *STN* subthalamic nuleus, *L* Left, *R* Right, – not applicable, *n.s.* non-significant, *s.e.* standard error.

### Emotional and behavioral problems and SN, STN and RN development

**Fig. 5:**
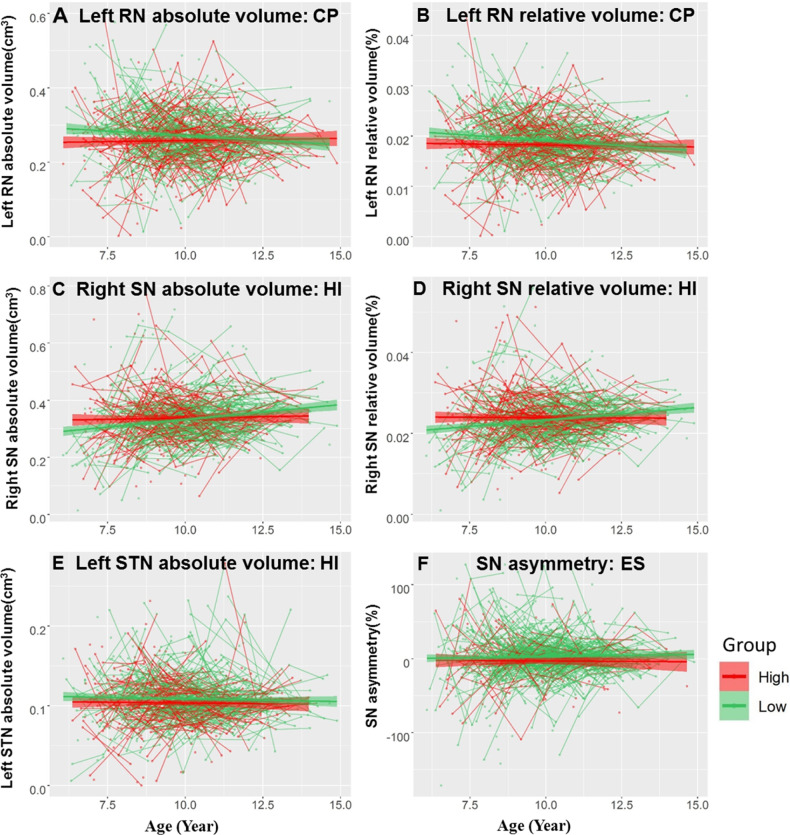
The different developmental trajectories of RN, SN, and STN volumes between relatively high-risk and relatively low-risk groups of emotional and behavioral problems. For visualization purposes, the sample was split into two groups: relatively high (red) and relatively low (green) emotional and behavioral problems. **A** The different developmental trajectories of left RN absolute volume on CP; **B** left RN relative volume on CP; **C** right SN absolute volume on HI; **D** right SN relative volume on HI; **E** left STN absolute volume on HI; **F** SN asymmetry on ES. Note that the statistical analyses were performed using a continuous emotional and behavioral problems score, and by adding this score as an interaction term to the best fitting model. Volumes and asymmetry (*y*-axis, cm^3^ for absolute volume and % for relative volume) by age (*x*-axis, years) are shown and the shaded areas represent the 95% confidence intervals. RN red nucleus, SN substantia nigra, STN subthalamic nucleus, ES Emotional symptoms, CP Conduct problems, HI hyperactivity/Inattention.

To investigate how children’s emotional and behavioral problems affect the development of the SN, STN and RN, we added these continuous scores as an interaction term to the best fitting model (Table S2 and Fig. 5). In addition, in order to properly interpret the results, we also mapped the developmental trajectories of emotional and behavioral problems, and the results showed that emotional symptoms, conduct problems and hyperactivity/inattention decreased with age, while peer relationship problems and prosocial behaviors did not show a trend with age. Details are in Table S3.

#### Emotional symptoms change the direction of SN lateralization

The asymmetry of the SN showed a significant main effect on emotional symptoms (*standardized β* = −0.072, *p* = 0.013; Fig. 5F), indicating that the greater the risk of emotional problems is, the smaller the lateralization. Although neither the high-risk (*standardized β* = −0.020, *p* = 0.78) nor low-risk (*standardized β* = 0.026, *p* = 0.41) groups for emotional symptoms showed a significant linear effect, it can be seen from Fig. 5F that the SN asymmetry was stronger in the low-risk group (mean = 2.85 ± 32.17) than in the high-risk group (mean = −3.02 ± 29.89) across the entire age group. To explore the reasons for the lateralization difference, we analyzed the difference in SN volume on the left and right sides between the high- and low-risk groups, and the results showed that the lateralization difference was mainly caused by the smaller volume on the right side of the high-risk group (left: low-risk: mean = 0.324 ± 0.077, high-risk: mean = 0.329 ± 0.076, *t* = −0.826, *p* = 0.410; right: low-risk: mean = 0.337 ± 0.089, high-risk: mean = 0.324 ± 0.094, *t* = 2.001, *p* = 0.046).

#### Conduct problems impede the RN volume from decreasing

A significant main effect on conduct problems in the absolute volume of the left RN (Fig. 5A) and a significant age × conduct symptoms interaction in the relative volumes of the left RN were found (Fig. 5B). Group analysis showed that the high-risk group exhibited a smaller absolute volume (high-risk: 0.258 ± 0.076; low-risk: 0.272 ± 0.078) and a slower decline in relative volume than the low-risk group (high-risk: *standardized β* = −0.023, *p* = 0.60; low-risk: *standardized β* = −0.129, *p* = 9.30 × 10^−4^) in the left RN.

#### Hyperactivity/inattention weakens the increase in SN volume and reduces the absolute volume of the STN

Significant age×hyperactivity/inattention interactions were found in the absolute and relative volumes of the right SN (Fig. 5C, D). Group analysis found that the high-risk group showed weaker increases in both absolute (high-risk: *standardized β* = 0.009, *p* = 0.85; low-risk: *standardized β* = 0.248 × 10^−2^, *p* = 4.24 × 10^−9^) and relative (high-risk: *standardized β* = −0.016, *p* = 0.73; low-risk: *standardized β* = 0.187, *p* = 4.00 × 10^−7^) volume than the low-risk group in the right SN.

The absolute volume of the left STN showed a significant main effect on hyperactivity/inattention (*standardized β* = −0.084, *p* = 6.00 × 10^−3^; Fig. 5E), indicating that the greater the risk of hyperactivity/inattention is, the smaller the absolute volume of the left STN. Although neither the high-risk (*standardized β* = −0.041, *p* = 0.39) nor low-risk (*standardized β* = −0.050, *p* = 0.18) group for emotional symptoms showed a significant linear effect, it can be seen from Fig. 5E that the absolute volume of the left STN was greater in the low-risk group (mean = 0.109 ± 0.031) than in the high-risk group (mean = 0.104 ± 0.033) across the entire age group.

## Discussion

In this study, we explored the developmental trajectories and behavior correlations of the SN, STN and RN in a four-year follow-up cohort study. To our knowledge, this is the first study to map the developmental trajectories of the SN, STN and RN using a longitudinal cohort. In addition, we found that emotional and behavioral problems, especially emotional symptoms, conduct problems, and hyperactivity/inattention, change their developmental trajectories from childhood to adolescence.

### The volume of the RN showed an opposite developmental trend to that of the SN and STN, which was affected by intracranial volume rather than gender

The absolute and relative volumes of the bilateral SN and right STN showed linear increases, while the absolute volume of the right RN and relative volume of the bilateral RN decreased linearly. Although the developmental trajectories of the volume and asymmetry of the SN, STN and RN have not been mapped in the past, the reasons for the results in this study can be inferred from previous studies. Since the structure of the SN can be easily determined by transcranial ultrasound, this technique has been used in most of the early quantitative measurements of the structure of the SN. Currently, two studies have investigated the developmental trajectory of SN during childhood and adolescence. One study with children aged 0–192 months showed a significant decrease in SN echogenicity with age [13], while another study with ultrasound aged 7–16 years showed no age effect [14]. The finding of the former is contrary to the findings of the present study, which may due to the differences in study indicators. Higher SN echogenicity is associated with lower dopaminergic cell numbers [13], while the association for SN volume is the opposite. Therefore, the linear rise in SN volume found in the present study represents the same developmental process as the decline in ultrasonic echogenicity. Regarding why the latter study failed to find an age effect, we think there may be two reasons. First, the sample size used in this study was too small to detect a significant age effect. Second, the study samples were half healthy individuals and half individuals with ADHD. ADHD itself can enhance echogenicity [14], which is exactly the opposite of the linear decline of the age effect and prevents the significance of the age effect.

The absolute and relative volumes of the STN showed linear increases in the right hemisphere but no change in the left hemisphere, which also led to an increase in right lateralization with age. The STN is highly connected to the SN, and they are affected in neurodegenerative diseases [42]. The SN is a dopamine nucleus, and animal models have confirmed that pathological changes in the STN are mainly related to changes in dopamine [43]. Further studies also found that the STN compensates for pathological changes in the SN, such as the loss of SN pathologic dopamine cells by enhancing its connection with cortical regions [44]. All these findings provide an important basis for the structural covariation of the STN and SN, and this study further demonstrated that the STN and SN also showed a similar linear rise in the development trajectory from childhood to adolescence.

The right lateralization development of the STN was first observed during childhood and adolescence in this study, which was caused by a rapid linear rise in the right STN and an insignificant linear rise in the left STN. There may be two reasons for the slow development of the left STN. First, because the development of the left STN lags behind that of the right STN, the volume of the left STN is significantly smaller than that of the right STN. Second, because the left STN develops earlier than the right STN, the development becomes slows down in childhood and adolescence, and it is difficult to detect a significant linear rise. If so, the volume of the left STN is significantly larger than that of the right STN. In this study, we observed that the left STN was significantly larger than the right STN in both absolute (left: mean = 0.1068 ± 0.0318, right: mean = 0.0956 ± 0.0305, *t* = 10.72, *p* = 1.32 × 10^−25^) and relative volume (left: mean = 0.0075 ± 0.0022, right: mean = 0.0067 ± 0.0021, *t* = 10.92, *p* = 1.51 × 10^−26^). This suggests that the right lateralization development of the STN in childhood and adolescence may be caused by the earlier development of the left STN.

The developmental trajectory of RN exhibited an inverse pattern compared to that of STN and SN, that is, the absolute volume of the right RN and the relative volume of the bilateral RN showed a linear decline. Previous studies based on Parkinson’s disease(PD) patients have found that the volume variation directions of the SN, STN and RN are often opposite [30], which may be related to the compensatory mechanism of the RN for the SN [4]. When SN dopamine neurons are lost, to ensure that function is not impaired, the RN volume increases and compensates for the SN through the thalamo-rubro-cerebellar pathway [4]. Studies have found that the volume of early-onset PD is larger than that of late-onset PD, indicating that early-onset PD opens the compensatory mechanism of RN earlier. An analysis of postmortem brains revealed that the RN of Parkinson’s patients was 32% larger than that of healthy controls. Therefore, the decrease in RN volume in this study may be related to the increase in SN volume and enhanced function. This makes the compensatory region of the RN shrink, providing sufficient space for compensatory growth after the loss of SN dopamine cells.

Previous studies have confirmed that the intracranial volume shows significant increases throughout childhood and adolescence [45, 46]. The intracranial volume is an important factor in the study of brain structure changes. For example, changes in the same direction as intracranial volume may exaggerate the effect of brain structure changes, while changes in the opposite direction may obscure the effect easily. In this study, the SN and STN both increased linearly, consistent with the development and change trend of intracranial volume. Therefore, it can be clearly seen that the growth trend of absolute volumes of the SN and STN were significantly larger than that of relative volume. However, since the RN was contrary to the change trend of intracranial volume, the linear decrease in the left RN was obscured, and the linear rise of the absolute volume was not significant. The linear increase in relative volume that excluded the intracranial volume effect reached a significant level. In addition, gender is another important factor that affects the trajectory of brain structure; therefore, previous studies have found that the brains of girls mature earlier than those of boys [47, 48]. However, this study did not find an effect of gender on the developmental trajectory, which may be related to the area of action of sex hormones. For example, the three brain structures (the SN, STN and RN) in this study are more related to the number of dopamine cells, while sex hormones mainly act on GABAergic neurons and dopamine terminals in the striatum [49]. Therefore, the development of the striatum and related brain areas will be affected by gender factors [37, 50]. However, the SN, STN, and RN release or regulate the release of dopamine into the striatum dopamine terminal and therefore, are not affected by sex hormones.

### Hyperactivity/inattention weakens the increase in SN volume and reduces the absolute volume of the STN

The characteristic symptoms of ADHD, such as inattention, hyperactivity, and impulsivity, are thought to be related to dysfunction of the dopaminergic frontostriatum and mesolimbic circuits that are heavily dominated by the midbrain dopamine system [15]. The SN is an important midbrain dopamine nucleus, and approximately 68% of the dopaminergic neurons in the midbrain are in the SN [11]. Previous studies have found that the SN echogenicity of children with ADHD is higher than that of healthy children [14, 16, 51]; in addition, the increase in echogenicity is significantly correlated with inattention, hyperactivity and impulsivity [15], while the higher echogenicity of the SN corresponds to a smaller volume. These results suggest that ADHD symptoms may hinder the increase in SN volume. In this study, by drawing the SN growth curve, we found that the linear growth rate of the high-risk group with high ADHD symptoms was significantly lower than that of the low-risk group, which provided a more basic and dynamic biological indicator for the prevention of ADHD.

In addition, we found that the absolute volume of the left STN was smaller in the high-risk group than in the low-risk group across the age groups, but this difference disappeared in the relative volume, which ruled out the effect of intracranial volume. Previous studies have found that ADHD could lead to reduced intracranial volume [52, 53]. Therefore, we suggest that this reduction in absolute volume of the left STN may be caused by the overall reduction in intracranial volume in the high-risk group, rather than the specific reduction in the STN.

### Conduct problems impede the RN volume from decreasing

This study, for the first time, identified the influence of conduct problems on the developmental trajectory of RN. We observed that the group at high risk of conduct problems had a lower rate of decline in RN volume than the group at low risk. The conduct problem is one of the most common mental problems in children and adolescents, similar to other externalizing disorders characterized by impulsive and destructive behavior, and is associated with reduced reactivity of the striatum to rewards [54–56] and aberrant connectivity of the fronto-striatal pathways. Although no studies have found a relationship between the RN and conduct disorder, it has been found that the RN could compensate for the damaged striato-thalamo-cortical circuit through the thalamo-rubro-cerebellar pathway formed with the cerebellum and thalamus [4], and this compensation is mainly reflected in the increase in RN volume [30]. In this study, the rate of the reduction of the RN slowed in the high-risk conduct problems group, which may be to compensate for the fronto-striatal pathway damage caused by conduct problems.

### Emotional symptoms change the direction of SN lateralization

In this study, we found that right lateralization in the low emotional problems risk group was significantly greater than that in the high-risk group, and the lateralization in the high-risk group was negative, that is, left lateralization. To further explore the reasons for the lateralization difference, we analyzed the difference in SN volume on the left and right sides between the high- and low-risk groups, and the results showed that the difference was mainly caused by the reduction in the right SN volume in the high-risk group. Abnormal SN lateralization was first discovered in a study of Parkinson’s disease, and the researchers found that the left SN of Parkinson’s patients was different from that of the healthy group [31, 57]. Another study on traumatic brain injury found that the volume of the SN on the left side in individuals with traumatic brain injury was significantly smaller than that in the healthy group, and the functional connection between the left SN and the left angular gyrus was significantly related to emotional problems [10]. Although the findings of the above studies focus on the left SN, this is not inconsistent with the abnormal SN in the right side of the high-emotional risk group found in this study. The above studies all used cross-sectional data from small samples of adult or elderly patients. Although both Parkinson’s disease and traumatic brain injury present with left SN injury and emotional problems, it has not been confirmed that the structural damage to the left SN is caused by emotional problems.

## Limitations

The study has some shortcomings. First, the sample only included children/adolescents aged 6–15 years. We found a significant STN growth effect only on the right, not the left. Since the volume of the STN on the left side is significantly larger than that on the right side, it is speculated that the STN on the left side may develop earlier and slower in childhood and adolescence, so a significant age effect could not be detected. However, we did not have data before the age of six. Future studies could also include data from before age 6 to verify this. Second, previous studies have found that puberty is an important factor in the development of brain structure [58–60], but this study was unable to examine the relationship due to the lack of puberty data. Therefore, it is hoped that future studies can solve this problem by collecting data on puberty.

## Conclusions

In this study, we first mapped the developmental trajectories of the SN, STN and RN volumes and asymmetries from childhood to adolescence using a longitudinal cohort study, and then we explored in detail the association of emotional and behavioral problems with their trajectories. The results showed that the volumes of the SN and right STN and the symmetry of the STN increased linearly from childhood to adolescence, while the volume of the RN decreased linearly. In addition, hyperactivity/inattention weakened the increase in SN volume and reduced the absolute volume of the STN, conduct problems impeded the RN volume from decreasing, and the emotional symptoms changed the direction of SN lateralization. This study provides the first evidence of how emotional and behavioral problems affect the dynamic development of the SN, STN and RN, which provides an important basis and guidance for the prevention and intervention of cognitive and emotional behavioral problems.

### Supplementary information

Supplemental material

## Data Availability

The raw data supporting the conclusions of this article were from the Children School Functions and Brain Development Project (CBD, Beijing Cohort), which will be soon made public.

## References

[1] de Hollander G, Keuken MC, van der Zwaag W, Forstmann BU, Trampel R (2017). Comparing functional MRI protocols for small, iron-rich basal ganglia nuclei such as the subthalamic nucleus at 7 T and 3 T. Hum Brain Mapp.

[2] Sonne J, Reddy V, Beato MR Neuroanatomy, Substantia Nigra. *StatPearls*. StatPearls Publishing, 2021. 30725680

[3] Smith ID, Grace AA (1992). Role of the subthalamic nucleus in the regulation of nigral dopamine neuron activity. Synapse.

[4] Philippens IHCHM, Wubben JA, Franke SK, Hofman S, Langermans JAM (2019). Involvement of the red nucleus in the compensation of Parkinsonism may explain why primates can develop Stable Parkinson’s Disease. Sci Rep.

[5] Langley J, Hussain S, Flores JJ, Bennett IJ, Hu X (2020). Characterization of age-related microstructural changes in locus coeruleus and substantia nigra pars compacta. Neurobiol Aging.

[6] Cao CY, Pan YX, Li DY, Zhan SK, Zhang J, Sun BM (2013). Subthalamus deep brain stimulation for primary dystonia patients: a long-term follow-up study. Mov Disord.

[7] van Horne CG, Quintero JE, Slevin JT, Anderson-Mooney A, Gurwell JA, Welleford AS (2018). Peripheral nerve grafts implanted into the substantia nigra in patients with Parkinson’s disease during deep brain stimulation surgery: 1-year follow-up study of safety, feasibility, and clinical outcome. J Neurosurg.

[8] Tomasi D, Volkow ND (2014). Functional connectivity of substantia nigra and ventral tegmental area: maturation during adolescence and effects of ADHD. Cereb Cortex.

[9] Tan L, Ge H, Tang J, Fu C, Duanmu W, Chen Y (2015). Amantadine preserves dopamine level and attenuates depression-like behavior induced by traumatic brain injury in rats. Behav Brain Res.

[10] Gao L, Xue Q, Gong S, Li G, Tong W, Fan M (2022). Structural and functional alterations of substantia nigra and associations with anxiety and depressive symptoms following traumatic brain injury. Front Neurol.

[11] Hirsch EC, Mouatt A, Faucheux B, Bonnet AM, Agid Y (1992). Dopamine, tremor, and Parkinson’s disease. Lancet.

[12] Manjon JV, Berto A, Romero JE, Lanuza E, Vivo-Hernando R, Aparici-Robles F (2020). pBrain: A novel pipeline for Parkinson related brain structure segmentation. NeuroImage Clin.

[13] Iova A, Garmashov A, Androuchtchenko N, Kehrer M, Berg D, Becker G (2004). Postnatal decrease in substantia nigra echogenicity - Implications for the pathogenesis of Parkinson’s disease. J Neurol.

[14] Romanos M, Weise D, Schliesser M, Schecklmann M, Loffler J, Warnke A (2010). Structural abnormality of the substantia nigra in children with attention-deficit hyperactivity disorder. J psychiatry Neurosci : JPN.

[15] Krauel K, Feldhaus HC, Simon A, Rehe C, Glaser M, Flechtner HH (2010). Increased echogenicity of the substantia nigra in children and adolescents with attention-deficit/hyperactivity disorder. Biol Psychiatry.

[16] Sepehrmanesh Z, Asayeshi A, Kakhki RD, Assarian F, Rahimi H, Arani SRM (2023). Echogenicity and size of substantia nigra on transcranial sonography (TCS) in patients with attention-deficit/hyperactivity disorder and healthy children aged 6-12 years: a comparative study. Egypt J Neurol Psychiatry Neurosurg.

[17] Elliott BL, D’Ardenne K, Mukherjee P, Schweitzer JB, McClure SM (2022). Limbic and executive meso- and nigrostriatal tracts predict impulsivity differences in attention-deficit/hyperactivity disorder. Biol Psychiatry Cogn Neurosci Neuroimaging.

[18] Lambert C, Zrinzo L, Nagy Z, Lutti A, Hariz M, Foltynie T (2012). Confirmation of functional zones within the human subthalamic nucleus: Patterns of connectivity and sub-parcellation using diffusion weighted imaging. Neuroimage.

[19] de Hollander G, Keuken MC, Forstmann BU (2015). The subcortical cocktail problem; mixed signals from the subthalamic nucleus and substantia nigra. PLoS One.

[20] Duann JR, Ide JS, Luo X, Li CS (2009). Functional connectivity delineates distinct roles of the inferior frontal cortex and presupplementary motor area in stop signal inhibition. J Neurosci : Off J Soc Neurosci.

[21] Beauregard M, Levesque J (2006). Functional magnetic resonance imaging investigation of the effects of neurofeedback training on the neural bases of selective attention and response inhibition in children with attention-deficit/hyperactivity disorder. Appl Psychophysiol Biofeedback.

[22] Yu Y, FitzGerald TH, Friston KJ (2013). Working memory and anticipatory set modulate midbrain and putamen activity. J Neurosci : Off J Soc Neurosci.

[23] Yoon JH, Minzenberg MJ, Raouf S, D’Esposito M, Carter CS (2013). Impaired prefrontal-basal ganglia functional connectivity and substantia nigra hyperactivity in schizophrenia. Biol Psychiatry.

[24] Mansfield EL, Karayanidis F, Jamadar S, Heathcote A, Forstmann BU (2011). Adjustments of response threshold during task switching: a model-based functional magnetic resonance imaging study. J Neurosci : Off J Soc Neurosci.

[25] Espinosa-Parrilla JF, Baunez C, Apicella P (2013). Linking reward processing to behavioral output: motor and motivational integration in the primate subthalamic nucleus. Front Comput Neurosci.

[26] Eagle DM, Baunez C (2010). Is there an inhibitory-response-control system in the rat? Evidence from anatomical and pharmacological studies of behavioral inhibition. Neurosci Biobehav Rev.

[27] Beck AK, Lutjens G, Schwabe K, Dengler R, Krauss JK, Sandmann P (2018). Thalamic and basal ganglia regions are involved in attentional processing of behaviorally significant events: evidence from simultaneous depth and scalp EEG. Brain Struct Funct.

[28] Uslaner JM, Robinson TE (2006). Subthalamic nucleus lesions increase impulsive action and decrease impulsive choice - mediation by enhanced incentive motivation?. Eur J Neurosci.

[29] Jellinger KA (2008). Neuropathological aspects of Alzheimer disease, Parkinson disease and frontotemporal dementia. Neurodegener Dis.

[30] Colpan ME, Slavin KV (2010). Subthalamic and red nucleus volumes in patients with Parkinson’s disease: Do they change with disease progression?. Parkinsonism Relat Disord.

[31] Zhong Z, Merkitch D, Karaman MM, Zhang J, Sui Y, Goldman JG (2019). High-spatial-resolution diffusion MRI in Parkinson disease: lateral asymmetry of the substantia Nigra. Radiology.

[32] Goodman R (1997). The Strengths and Difficulties Questionnaire: a research note. J Child Psychol Psychiatry.

[33] Giraud R, Ta VT, Papadakis N, Manjon JV, Collins DL, Coupe P (2016). An Optimized PatchMatch for multi-scale and multi-feature label fusion. Neuroimage.

[34] Wang Y, Xu Q, Li S, Li G, Zuo C, Liao S (2018). Gender differences in anomalous subcortical morphology for children with ADHD. Neurosci Lett.

[35] Pinheiro JC, Bates DJ, Debroy SD, Sakar D (2009). NLME: Linear and Nonlinear Mixed Effects Models. R package version.

[36] Vijayakumar N, Mills KL, Alexander-Bloch A, Tamnes CK, Whittle S (2018). Structural brain development: A review of methodological approaches and best practices. Dev Cogn Neurosci.

[37] Raznahan A, Shaw PW, Lerch JP, Clasen LS, Greenstein D, Berman R (2014). Longitudinal four-dimensional mapping of subcortical anatomy in human development. Proc Natl Acad Sci USA.

[38] Paulsen DJ, Hallquist MN, Geier CF, Luna B (2015). Effects of incentives, age, and behavior on brain activation during inhibitory control: a longitudinal fMRI study. Dev Cogn Neurosci.

[39] Casey BJ (2015). Beyond simple models of self-control to circuit-based accounts of adolescent behavior. Annu Rev Psychol.

[40] Perneger TV (1998). What’s wrong with Bonferroni adjustments. BMJ.

[41] Sankoh AJ, Huque MF, Dubey SD (1997). Some comments on frequently used multiple endpoint adjustment methods in clinical trials. Stat Med.

[42] Hidding U, Gulberti A, Horn A, Buhmann C, Hamel W, Koeppen JA (2017). Impact of combined subthalamic nucleus and substantia nigra stimulation on neuropsychiatric symptoms in Parkinson’s disease patients. Parkinson’s Dis.

[43] Massey LA, Yousry TA (2010). Anatomy of the substantia nigra and subthalamic nucleus on MR imaging. Neuroimaging Clin North Am.

[44] Nambu A, Tokuno H, Takada M (2002). Functional significance of the cortico-subthalamo-pallidal ‘hyperdirect’ pathway. Neurosci Res.

[45] Mills KL, Goddings AL, Herting MM, Meuwese R, Blakemore SJ, Crone EA (2016). Structural brain development between childhood and adulthood: Convergence across four longitudinal samples. Neuroimage.

[46] Dhamala E, Ooi LQR, Chen J, Kong R, Anderson KM, Chin R (2022). Proportional intracranial volume correction differentially biases behavioral predictions across neuroanatomical features, sexes, and development. Neuroimage.

[47] Bramen JE, Hranilovich JA, Dahl RE, Forbes EE, Chen J, Toga AW (2011). Puberty influences medial temporal lobe and cortical gray matter maturation differently in boys than girls matched for sexual maturity. Cereb Cortex.

[48] Cole WR, Mostofsky SH, Larson JC, Denckla MB, Mahone EM (2008). Age-related changes in motor subtle signs among girls and boys with ADHD. Neurology.

[49] Becker JB (1999). Gender differences in dopaminergic function in striatum and nucleus accumbens. Pharmacol, Biochem, Behav.

[50] Wierenga L, Langen M, Ambrosino S, van Dijk S, Oranje B, Durston S (2014). Typical development of basal ganglia, hippocampus, amygdala and cerebellum from age 7 to 24. Neuroimage.

[51] Marcel R, David W, Martin S, Andreas W, Manfred G, Joseph C (2010). Structural alteration of substantia nigra in ADHD. Eur Child Adolesc Psychiatry.

[52] Durston S, Hulshoff Pol HE, Schnack HG, Buitelaar JK, Steenhuis MP, Minderaa RB (2004). Magnetic resonance imaging of boys with attention-deficit/hyperactivity disorder and their unaffected siblings. J Am Acad Child Adolesc Psychiatry.

[53] Castellanos FX (2021). A biased perspective on brain imaging of ADHD. Am J psychiatry.

[54] Sterzer P, Stadler C (2009). Neuroimaging of aggressive and violent behaviour in children and adolescents. Front Behav Neurosci.

[55] Holz NE, Boecker-Schlier R, Buchmann AF, Blomeyer D, Jennen-Steinmetz C, Baumeister S (2017). Ventral striatum and amygdala activity as convergence sites for early adversity and conduct disorder. Soc Cogn Affect Neurosci.

[56] Wallace GL, White SF, Robustelli B, Sinclair S, Hwang S, Martin A (2014). Cortical and subcortical abnormalities in youths with conduct disorder and elevated callous-unemotional traits. J Am Acad Child Adolesc Psychiatry.

[57] Scherfler C, Seppi K, Mair KJ, Donnemiller E, Virgolini I, Wenning GK (2012). Left hemispheric predominance of nigrostriatal dysfunction in Parkinson’s disease. Brain.

[58] Giedd JN, Clasen LS, Lenroot R, Greenstein D, Wallace GL, Ordaz S (2006). Puberty-related influences on brain development. Mol Cell Endocrinol.

[59] Goddings AL, Mills KL, Clasen LS, Giedd JN, Viner RM, Blakemore SJ (2014). The influence of puberty on subcortical brain development. Neuroimage.

[60] Hu S, Pruessner JC, Coupe P, Collins DL (2013). Volumetric analysis of medial temporal lobe structures in brain development from childhood to adolescence. Neuroimage.

