# Supplementary material for: Association of emotional and behavioral problems with the development of the substantia nigra, subthalamic nucleus, and red nucleus volumes and asymmetries from childhood to adolescence: A longitudinal cohort study
Source: Transl Psychiatry. 2024 Feb 26;14:117. doi: 10.1038/s41398-024-02803-4 (PMC10894865; doi:10.1038/s41398-024-02803-4)
Supplement: Supplementary file 1 — Supplemental material [file 41398_2024_2803_MOESM1_ESM.docx]

**Supplemental Information**

**Supplemental Methods**

**Sample selected**

We used a longitudinal structural MRI dataset comprising the data of 766 normally developing children aged 6-15 years (F/M = 334/432) from the Children School Functions and Brain Development project (CBD, Beijing Cohort). Participants underwent 2-4 repeated MRI scans at approximately one-year intervals, resulting in 1603 structural MRI scans in total. The exclusion criteria included notable physical illness or head trauma and a history of neurological/psychiatric disorders. None of the children had taken any drugs or consumed caffeine on the day of the behavioral tests and MRI scans. All participants were recruited from primary schools in Beijing and had normal cognitive ability; they were assessed with a well-validated Chinese standardized cognitive ability test [^1^](#_ENREF_1). The exclusion criteria included notable physical illness or head trauma and a history of neurological/psychiatric disorders. None of the children had taken any drugs or consumed caffeine on the day of the behavioral tests and MRI scans. One hundred seventy-three structural MRI scans were excluded due to artifacts after data quality control, and 204 structural MRI scans were removed because assessments of emotional and behavioral problems had not been conducted. Finally, structural MRI data of 667 children (6–15 years old at baseline, F/M = 307/360, 1226 scans in total) remained, with 4 scans were available for 36 children (F/M = 19/17), 3 scans available for 126 children (F/M = 63/63), 2 scans were available for 199 children (F/M = 85/114) and 1 scan available for 306 children (F/M = 140/166).

**MRI quality assurance**

All MRI scan quality control procedures are described below. i) Individual images were carefully visually examined by an experienced radiologist to rule out incidental abnormalities, such as neuroepithelial cysts, arachnoid cysts, and other intracranial space-occupying lesions. ii) Five experienced raters each performed careful visual inspections with a scan rating procedure, with a protocol similar to that of the Human Connectome Project [^2^](#_ENREF_2). iii) Images that received high quality ratings from all raters were retained.

**Statistical analysis**

**Criteria for defining high-low risk groups for emotional and behavioral problems**

To visualize and better show the role of emotional and behavioral problems, the sample was split into two subgroups by the median of emotional and behavioral problems [^3^](#_ENREF_3): relatively low (mean = 0.79, SD = 0.76, range = 0-2) and relatively high (mean = 3.80, SD = 0.98, range = 3-8) emotional symptoms, low (mean = 0.73, SD = 0.45, range = 0-1) and relatively high (mean = 2.62, SD = 0.89, range = 2-7) conduct problems, low (mean = 2.41, SD = 1.35, range = 0-4) and relatively high (mean = 6.42, SD = 1.38, range = 5-10) hyperactivity/inattention, low (mean = 1.45, SD = 1.02, range = 0-3) and relatively high (mean = 4.45, SD = 0.76, range=4-8) peer problems, and low (mean = 8.63, SD = 1.14, range=7-10) and relatively high (mean = 5.08, SD = 1.03, range=0-6) prosocial behaviors.

**Multiple comparison correction**

To optimally balance between Type-I and Type-II error, we took the correlation between the dependent variables into account by using a Bonferroni procedure adjusted for correlated variables (bilateral substantia nigra, red nucleus, and subthalamic nucleus) (<http://www.quantitativeskills.com/sisa/calculations/bonfer.htm>) [^4^](#_ENREF_4)^,^[^5^](#_ENREF_5). Using a Bonferroni correction that treats the variables as independent (proper Bonferroni: alpha/number of tests), would lead to a too stringent correction, as the dependent variables are not obtained in independent subgroups. Absolute volumes showed a mean correlation coefficient of r = 0.235, leading to an equivalent corrected alpha of 0.0127 (number of tests = 6). Relative volumes showed a mean correlation coefficient of r = 0.198, leading to an equivalent corrected alpha of 0.0119 (number of tests = 6). Asymmetries showed a mean correlation coefficient of r = 0.229, leading to an equivalent corrected alpha of 0.0214 (number of tests = 3).

**References**

1 Dong, Q. & Lin, C. D. *Standardized tests in children and adolescent mental development in China*. (Beijing: science press, 2011).

2 Marcus, D. S. *et al.* Human Connectome Project informatics: quality control, database services, and data visualization. *Neuroimage* **80**, 202-219, doi:10.1016/j.neuroimage.2013.05.077 (2013).

3 Tamnes, C. K., Bos, M. G. N., van de Kamp, F. C., Peters, S. & Crone, E. A. Longitudinal development of hippocampal subregions from childhood to adulthood. *Dev Cogn Neurosci* **30**, 212-222, doi:10.1016/j.dcn.2018.03.009 (2018).

4 Perneger, T. V. What's wrong with Bonferroni adjustments. *BMJ* **316**, 1236-1238, doi:10.1136/bmj.316.7139.1236 (1998).

5 Sankoh, A. J., Huque, M. F. & Dubey, S. D. Some comments on frequently used multiple endpoint adjustment methods in clinical trials. *Statistics in Medicine* **16**, 2529-2542, doi:10.1002/(sici)1097-0258(19971130)16:223.0.co;2-j (1997).

**Supplemental Figures**


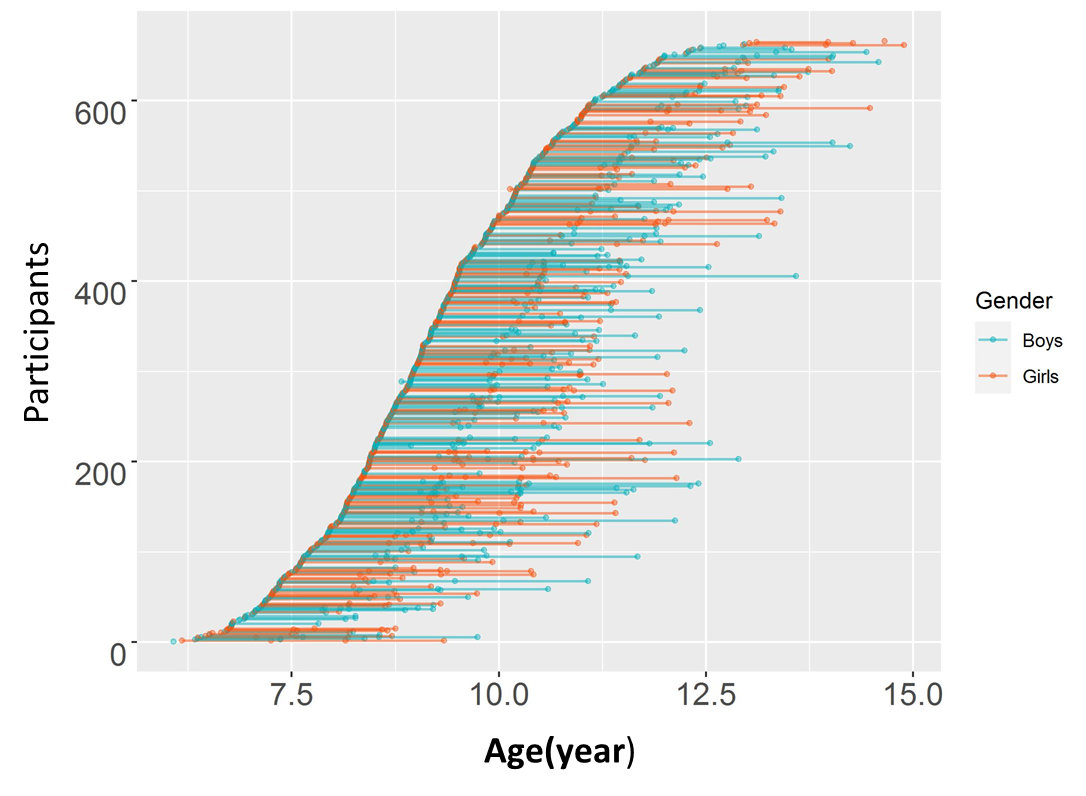


Figure S1. Distribution of ages for subjects included in the current study.

**Supplemental Tables**

Table S1 BIC values for the comparison of different mixed models examining age and sex effect on absolute, relative volumes, asmmetries of the RN, SN, STN

| Volumes | | | Random intercept | Age:Linear | Age: Quadractic | Age: Cubic | Random slope | Sex main effect | Sex Interaction effect |
| --- | --- | --- | --- | --- | --- | --- | --- | --- | --- |
| Absolute volumes | RN | L | **-2784.458** | -2783.481 | -2765.121 | -2744.243 | -2778.306 | -2764.691 | - |
|  |  | R | -3011.856 | **-3047.425** | -3021.126 | -3000.107 | -3040.912 | -3034.785 | -3018.026 |
|  | SN | L | -2771.350 | -2822.598 | -2802.666 | -2782.232 | -2810.992 | **-2828.814** | -2813.540 |
|  |  | R | -2419.665 | -2459.172 | -2444.112 | -2428.567 | -2456.785 | **-2465.035** | -2449.676 |
|  | STN | L | **-4963.725** | -4962.885 | -4941.633 | -4919.032 | -4951.403 | -4947.197 | - |
|  |  | R | -5002.907 | **-5047.867** | -5032.431 | -5014.113 | -5035.531 | -5031.594 | -5014.597 |
| Relative volumes | RN | L | -9262.327 | -9291.820 | -9274.038 | -9247.440 | -9294.613 | **-9296.926** | -9269.500 |
|  |  | R | -9547.954 | -9576.873 | -9556.072 | -9530.283 | -9561.536 | **-9581.090** | -9554.512 |
|  | SN | L | -9374.707 | **-9424.988** | -9399.621 | -9375.037 | -9415.426 | 9406.581 | -9384.520 |
|  |  | R | -8986.586 | **-9025.073** | -9004.162 | -8985.357 | -9003.295 | -9004.590 | -8982.845 |
|  | STN | L | **-11468.710** | -11503.180 | -11476.330 | -11449.180 | -11458.050 | -11462.260 | - |
|  |  | R | -11551.040 | -11577.320 | -11565.710 | -11543.880 | -11563.400 | **-11587.060** | -11553.920 |
| Asymmetries | RN | | **11894.430** | 11898.690 | 11905.960 | 11913.910 | 11915.370 | 11917.280 | - |
|  | SN | | **11985.440** | 11991.050 | 11996.000 | 12003.000 | 12016.470 | 12006.910 | - |
|  | STN | | 12616.640 | **12599.290** | 12605.010 | 12612.890 | 12602.740 | 12602.300 | 12606.690 |

RN = red nucleus, SN = substantia nigra, STN = subthalamic nuleus, L = Left, R = Right. Bold indicate the best model.

Table S2 Model parameters for fixed effects when including level of emotional and behavioural problems in the best fitting models for absolute, relative volumes, asmmetries of the RN, SN, STN.

| Volumes | | HS | Best fitting model | EBP Scales | Intercept(s.e.) | Site coefficient *β*_1_(s.e.) | Gender coefficient  *β*_2_(s.e.) | Age coefficient  *β*_3_(s.e.) | EBP coefficient  *β*_4_ (s.e.) | Age×EBP coefficient  *β*_5_(s.e.) |
| --- | --- | --- | --- | --- | --- | --- | --- | --- | --- | --- |
| Absolute volumes | RN | L | None | ES | 2.91(0.16)×10^-1^ | n.s. | -. | n.s. | n.s. | - |
|  |  |  |  | CP | 2.95(0.16)×10^-1^ | n.s. | - | n.s. | -5.17(1.96)×10^-3^ | - |
|  |  |  |  | HI | 2.95(0.17)×10^-1^ | n.s. | - | n.s. | n.s. | - |
|  |  |  |  | PP | 2.89(0.16)×10^-1^ | n.s. | - | n.s. | n.s. | - |
|  |  |  |  | PB | 2.69(0.19)×10^-1^ | n.s. | - | n.s. | n.s. | - |
|  |  | R | Linear | ES | 3.00(0.19)×10^-1^ | n.s. | - | n.s. | n.s. | n.s. |
|  |  |  |  | CP | 3.49(0.23)×10^-1^ | n.s. | - | -7.22(2.15)×10^-3^ | n.s. | n.s. |
|  |  |  |  | HI | 3.25(0.24)×10^-1^ | n.s. | - | n.s. | n.s. | n.s. |
|  |  |  |  | PP | 3.12(0.22)×10^-1^ | n.s. | - | n.s. | n.s. | n.s. |
|  |  |  |  | PB | 2.38(0.49)×10^-1^ | n.s. | - | n.s. | n.s. | n.s. |
|  | SN | L | Linear | ES | 2.42(0.20)×10^-1^ | n.s. | 2.39(0.49)×10^-2^ | 7.24(1.85)×10^-3^ | n.s. | n.s. |
|  |  |  |  | CP | 2.87(0.24)×10^-1^ | n.s. | 2.36(0.49)×10^-2^ | n.s. | n.s. | n.s. |
|  |  |  |  | HI | 2.59(0.26)×10^-1^ | n.s. | 2.36(0.49)×10^-2^ | n.s. | n.s. | n.s. |
|  |  |  |  | PP | 2.85(0.23)×10^-1^ | n.s. | 2.30(0.49)×10^-2^ | n.s. | n.s. | n.s. |
|  |  |  |  | PB | 2.55(0.54)×10^-1^ | n.s. | 2.26(0.49)×10^-2^ | n.s. | n.s. | n.s. |
|  |  | R | Linear | ES | 2.70(0.24)×10^-1^ | n.s. | 2.79(0.61)×10^-2^ | 6.89(2.16)×10^-3^ | n.s. | n.s. |
|  |  |  |  | CP | 3.07(0.28)×10^-1^ | n.s. | 2.92(0.61)×10^-2^ | n.s. | n.s. | n.s. |
|  |  |  |  | HI | 1.76(0.31)×10^-1^ | n.s. | 2.90(0.61)×10^-2^ | 1.57(0.29)×10^-2^ | 2.34(0.65)×10^-2^ | -2.34(0.66)×10^-3^ |
|  |  |  |  | PP | 2.88(0.27)×10^-1^ | n.s. | 2.86(0.61)×10^-2^ | n.s. | n.s. | n.s. |
|  |  |  |  | PB | 3.09(0.62)×10^-1^ | n.s. | 2.77(0.61)×10^-2^ | n.s. | n.s. | n.s. |
|  | STN | L | None | ES | 1.18(0.07)×10^-1^ | n.s. | - | n.s. | n.s. | - |
|  |  |  |  | CP | 1.19(0.07)×10^-1^ | n.s. | - | n.s. | n.s. | - |
|  |  |  |  | HI | 1.23(0.07)×10^-1^ | n.s. | - | n.s. | -1.13(0.41)×10^-3^ | - |
|  |  |  |  | PP | 1.15(0.07)×10^-1^ | n.s. | - | n.s. | n.s. | - |
|  |  |  |  | PB | 1.20(0.08)×10^-1^ | n.s. | - | n.s. | n.s. | - |
|  |  | R | Linear | ES | 7.67(0.81)×10^-2^ | n.s. | - | 2.21(0.75)×10^-3^ | n.s. | n.s. |
|  |  |  |  | CP | 9.36(0.99)×10^-2^ | n.s. | - | n.s. | n.s. | n.s. |
|  |  |  |  | HI | 7.80(1.07)×10^-2^ | n.s. | - | n.s. | n.s. | n.s. |
|  |  |  |  | PP | 8.33(0.94)×10^-2^ | n.s. | - | n.s. | n.s. | n.s. |
|  |  |  |  | PB | 1.02(0.22)×10^-1^ | n.s. | - | n.s. | n.s. | n.s. |
| Relative volumes | RN | L | Linear | ES | 2.36(0.14)×10^-2^ | n.s. | -1.42(0.34)×10^-3^ | -3.93(1.31)×10^-4^ | n.s. | n.s. |
|  |  |  |  | CP | 2.61(0.17)×10^-2^ | n.s. | -1.35(0.34)×10^-3^ | -6.19(1.65)×10^-4^ | -2.48(0.84)×10^-3^ | 2.24(0.84)×10^-4^ |
|  |  |  |  | HI | 2.38(0.19)×10^-2^ | n.s. | -1.38(0.34)×10^-3^ | n.s. | n.s. | n.s. |
|  |  |  |  | PP | 2.39(0.16)×10^-2^ | n.s. | -1.38(0.34)×10^-3^ | -4.17(1.56)×10^-4^ | n.s. | n.s. |
|  |  |  |  | PB | 1.61(0.38)×10^-2^ | n.s. | -1.31(0.34)×10^-3^ | n.s. | n.s. | n.s. |
|  |  | R | Linear | ES | 2.31(0.13)×10^-2^ | n.s. | -1.36(0.33)×10^-3^ | -2.98(1.18)×10^-4^ | n.s. | n.s. |
|  |  |  |  | CP | 2.60(0.16)×10^-2^ | n.s. | -1.33(0.33)×10^-3^ | -5.64(1.48)×10^-4^ | n.s. | n.s. |
|  |  |  |  | HI | 2.44(0.17)×10^-2^ | n.s. | -1.36(0.33)×10^-3^ | -4.39(1.59)×10^-4^ | n.s. | n.s. |
|  |  |  |  | PP | 2.36(0.15)×10^-2^ | n.s. | -1.36(0.33)×10^-3^ | -3.57(1.40)×10^-4^ | n.s. | n.s. |
|  |  |  |  | PB | 1.96(0.34)×10^-2^ | n.s. | -1.35(0.33)×10^-3^ | n.s. | n.s. | n.s. |
|  | SN | L | Linear | ES | 1.93(0.13)×10^-2^ | n.s. | - | 3.81(1.25)×10^-4^ | n.s. | n.s. |
|  |  |  |  | CP | 2.18(0.16)×10^-2^ | n.s. | - | n.s. | n.s. | n.s. |
|  |  |  |  | HI | 1.99(0.18)×10^-2^ | n.s. | - | n.s. | n.s. | n.s. |
|  |  |  |  | PP | 2.17(0.16)×10^-2^ | n.s. | - | n.s. | n.s. | n.s. |
|  |  |  |  | PB | 2.12(0.36)×10^-2^ | n.s. | - | n.s. | n.s. | n.s. |
|  |  | R | Linear | ES | 2.14(0.16)×10^-2^ | n.s. | - | n.s. | n.s. | n.s. |
|  |  |  |  | CP | 2.38(0.20)×10^-2^ | n.s. | - | n.s. | n.s. | n.s. |
|  |  |  |  | HI | 1.45(0.21)×10^-2^ | n.s. | - | 9.80(2.00)×10^-4^ | 1.70(0.45)×10^-3^ | -1.61(0.45)×10^-4^ |
|  |  |  |  | PP | 2.23(0.19)×10^-2^ | n.s. | - | n.s. | n.s. | n.s. |
|  |  |  |  | PB | 2.49(0.43)×10^-2^ | n.s. | - | n.s. | n.s. | n.s. |
|  | STN | L | None | ES | 9.07(0.46)×10^-3^ | n.s. | -5.92(1.43)×10^-4^ | n.s. | n.s. | - |
|  |  |  |  | CP | 9.13(0.46)×10^-3^ | n.s. | -5.83(1.43)×10^-4^ | n.s. | n.s. | - |
|  |  |  |  | HI | 9.33(0.48)×10^-3^ | n.s. | -5.68(1.43)×10^-4^ | n.s. | n.s. | - |
|  |  |  |  | PP | 8.91(0.46)×10^-3^ | n.s. | -6.03(1.43)×10^-4^ | n.s. | n.s. | - |
|  |  |  |  | PB | 9.26(0.52)×10^-3^ | n.s. | -6.02(1.44)×10^-4^ | n.s. | n.s. | - |
|  |  | R | Linear | ES | 6.12(0.56)×10^-3^ | n.s. | -4.90(1.33)×10^-4^ | n.s. | n.s. | n.s. |
|  |  |  |  | CP | 7.14(0.68)×10^-3^ | n.s. | -4.80(1.33)×10^-4^ | n.s. | n.s. | n.s. |
|  |  |  |  | HI | 6.02(0.73)×10^-3^ | n.s. | -4.78(1.33)×10^-4^ | n.s. | n.s. | n.s. |
|  |  |  |  | PP | 6.51(0.64)×10^-3^ | n.s. | -4.93(1.33)×10^-4^ | n.s. | n.s. | n.s. |
|  |  |  |  | PB | 8.08(1.50)×10^-3^ | n.s. | -5.08(1.34)×10^-4^ | n.s. | n.s. | n.s. |
| Asymmetries | RN | | None | ES | n.s. | n.s. | - | n.s. | n.s. | - |
|  |  |  |  | CP | n.s. | n.s. | - | n.s. | n.s. | - |
|  |  |  |  | HI | n.s. | n.s. | - | n.s. | n.s. | - |
|  |  |  |  | PP | n.s. | n.s. | - | n.s. | n.s. | - |
|  |  |  |  | PB | 1.68(0.70)×10 | n.s. | - | n.s. | n.s. | - |
|  | SN | | None | ES | n.s. | n.s. | - | n.s. | -1.66(0.67) | - |
|  |  |  |  | CP | n.s. | n.s. | - | n.s. | n.s. | - |
|  |  |  |  | HI | n.s. | n.s. | - | n.s. | n.s. | - |
|  |  |  |  | PP | n.s. | n.s. | - | n.s. | n.s. | - |
|  |  |  |  | PB | n.s. | n.s. | - | n.s. | n.s. | - |
|  | STN | | Linear | ES | -4.11(1.09)×10 | n.s. | - | 3.09(1.02) | n.s. | n.s. |
|  |  |  |  | CP | n.s. | n.s. | - | n.s. | n.s. | n.s. |
|  |  |  |  | HI | -4.74(1.45)×10 | n.s. | - | 3.55(1.38) | n.s. | n.s. |
|  |  |  |  | PP | n.s. | n.s. | - | n.s. | n.s. | n.s. |
|  |  |  |  | PB | n.s. | n.s. | - | n.s. | n.s. | n.s. |

The level of significance is 0.0127 for absolute volumes, 0.0119 for relative volumes and 0.0214 for asymmetry after multiple comparisons correction, – = not applicable, n.s. = non-significant, s.e. = standard error, HS = Hemisphere, L = left, R = right, RN = red nucleus, SN = substantia nigra, STN = subthalamic nuleus, EBP = emotional and behavioural problems, ES = Emotional Symptoms, CP =Conduct Problems, HI = Hyperactivity/Inattention, PP = Peer Relationship Problems, PB = Prosocial Behaviors.

Table S3 Best fit regression model for absolute, relative volumes, asymmetry of the RN, SN, STN and parameters for developmental trajectories.

| EBP | Best fitting model | Intercept (s.e.) | Site coefficient  *β*_1_ (s.e.) | Gender coefficient  *β*_2_ (s.e.) | Age coefficient  *β*_3_ (s.e.) | Age×gender  coeffiicent *β*_4_ (s.e.) |
| --- | --- | --- | --- | --- | --- | --- |
| ES | Linear | 2.42(0.28) | n.s. | - | -1.17(0.25) ×10^-1^ | - |
| CP | Linear | 1.98(0.23) | n.s. | - | -4.95(2.09) ×10^-2^ | - |
| HI | Linear | 6.27(0.45) | n.s. | - | -2.44(0.39) ×10^-1^ | - |
| PP | None | - | - | - | - | - |
| PB | None | - | - | - | - | - |

EBP = emotional and behavioural problems, ES = Emotional Symptoms, CP =Conduct Problems, HI = Hyperactivity/Inattention, PP = Peer Relationship Problems, PB = Prosocial Behaviors, – = not applicable, n.s. = non-significant, s.e. = standard error.
